# “I’m On My Own, I Need Support”: Needs Assessment of Community Aged Care Services

**DOI:** 10.5334/ijic.7005

**Published:** 2023-09-22

**Authors:** Genevieve Z. Steiner-Lim, Diana Karamacoska, Gamze Abramov, Shamieka Dubois, Anne Harley, Keith McDonald, Mark I. Hohenberg

**Affiliations:** 1 NICM Health Research Institute and Translational Health Research Institute (THRI), Western Sydney University, Penrith NSW 2751, AU; 2 NICM Health Research Institute, Western Sydney University, Penrith NSW 2751, AU; 3 School of Psychology, University of Wollongong, Wollongong NSW 2522, AU; 4 South Western Sydney Primary Health Network, Campbelltown NSW 2560, AU; 5 School of Medicine, Western Sydney University, Penrith NSW 2751, AU

**Keywords:** needs assessment, aged care, integrated care, community care, dementia, qualitative, geriatrics

## Abstract

**Introduction:**

Well-integrated community aged care services empower and enable older people to live and thrive in the community by supporting activities of daily living. To inform integrated community aged care service planning and delivery in South Western Sydney Australia, a needs assessment with consumers (i.e., older people), their caregivers, and healthcare providers was conducted. This study details the comprehensive and inclusive needs assessment process undertaken, with a focus on translating the findings into practice to improve integrated care.

**Description:**

Qualitative interviews and community forum-style focus groups engaged 160 stakeholders including GPs, older people, and aged care workers. Transcribed data were thematically coded using an inductive approach. Data were organised into four themes: 1) access to community aged care services; (2) healthcare and medical needs; (3) social concerns and needs; and (4) education and information needs.

**Discussion:**

The needs assessment undertaken identified unmet needs, gaps in service provision, and recommendations for improving integrated community aged care services.

**Conclusion:**

Findings are novel in the context of South Western Sydney, Australia. The study design, methods employed, and lessons learned can be adapted internationally for future needs assessments to inform policy, strategies, and integrated aged care service delivery.

## Introduction

Community aged care services aim to support older people with activities of daily living (ADLs), promoting inclusivity by enabling older people to live and prosper in the community [1]. Community aged care policy reforms have shifted to a model of consumer directed care, advocating for older people to move between services to meet their needs and preferences. Consumer directed care promotes personalised choices and flexibility and places the responsibility on the community aged care sector to provide suitable and inclusive options for diverse consumers in an integrated fashion [2,3,4].

Needs assessments that identify problems and solutions, including perspectives from a broad range of stakeholders and communities, are required to holistically inform service planning for consumer directed models of care [5,6]. Well-integrated interdisciplinary care that meets the multiple and complex healthcare needs of older people requires cross-sector and multi-organisational delivery to avoid fragmentation and unmet population needs, regardless of the international context [7]. Bradshaw’s taxonomy of social need encompasses normative, felt, expressed, and comparative needs [8]. Identifying the ‘felt needs’ (i.e., what people state they need) places stakeholders at the centre of an action-oriented advocacy process for change [9,10]. By canvassing the opinions of key stakeholders including the community and health professionals (‘normative needs’; i.e., professional defined desirable standards), findings can be triangulated, providing a more comprehensive and less biased picture of perceived needs and gaps in healthcare service provision as ‘expressed needs’ (i.e., felt needs turned into action) [8,11,12]. The ‘comparative needs’ (i.e., similar needs between locations) can be ascertained by conducting needs assessments across broad and diverse geographical regions [8].

South Western Sydney (SWS) is one of the most rapidly growing and diverse regions in Australia (see Supplementary Materials), with previous work [13] identifying fragmented and uncoordinated aged care between primary, acute, and community settings. The integrated care case study presented here showcases the application of a comprehensive needs assessment that highlights the perceived needs of many voices of the community including older people, caregivers, and healthcare providers. The long-term aim of the needs assessment was to inform the planning and implementation of consumer driven models of integrated community aged care services that cater for diverse communities.

## Needs Assessment Process

The needs assessment included 160 stakeholders in each of the seven local government areas (LGAs) of SWS: City of Canterbury-Bankstown, Camden Council, Campbelltown City Council, Fairfield City, Liverpool City Council, Wingecarribee Shire, and Wollondilly Shire Council. A qualitative approach using a combination of interviews, focus groups, and written feedback from forum attendees was taken to not restrict responses to normative needs.

To ensure that the needs assessment was comprehensive and representational, three stakeholder groups were involved:

1. GPs
2. community aged care and health workers
3. older people and caregivers.

The research team completed in-depth 30-minute telephone interviews with GPs, and facilitated separate 2-hour community forum-style focus groups with the other two groups hosted in community settings (libraries and community halls) within each of the seven LGAs (i.e., a total of 14 focus groups). Recruitment and running of the community forums took place over a 3-month time frame (February 2018–April 2018) and GPs over a 21-month time frame (February 2018–November 2019). A range of recruitment strategies were used: study flyers that were distributed by email via the SWS Primary Health Network (SWSPHN) and SWS Local Health District (SWSLHD), at continuing professional development events for GPs, by consumer and healthcare networks and social media, and the study was promoted online and in the LGAs’ newspapers. Ethical approval was obtained from SWSLHD Human Research Ethics Committee (HE17/265) and Western Sydney University Human Research Ethics Committee (H12417) and only participants providing written informed consent and the research team were present during data collection.

Our research team facilitators used a semi-structured interview question guide to pose a series of open-ended questions to participants to ascertain their understanding of the existing aged care service provision for older people in their communities (see Supplementary Materials). The guide was structured into themes to identify the healthcare, medical, social, education, and information needs of older people and caregivers and from these, to determine priority areas for future healthcare provision planning.

### Data analysis

All GP interviews and community forums were audio recorded and transcribed verbatim. Any potentially identifiable information was removed, and pseudonyms were used throughout. Transcripts from all groups/data sources underwent thematic (inductive) analysis. Transcripts were coded in Quirkos v.1.4.1 software taking an inductive approach with the method of constant comparison. This iterative process consisted of systematically identifying, comparing and coding themes within and between the data sources by two members of the research team [14]. Emerging categories and associations among the codes led to the development of several themes and sub-themes. Our team’s previous needs assessments have used similar qualitative methodologies in this region of Australia [6,13].

## Results

### Study population

Participant group numbers across LGAs are detailed in Table 1. The total sample size recruited (*N* = 160) exceeded the target sample size (*N* = 140; 20 participants × 7 LGAs), with the desired sample size of 15–20 participants achieved for each LGA except for Campbelltown, despite repeated recruitment efforts in this area. In addition to transcriptions, follow-up written feedback was received via email from forum attendees: older people/caregivers in Wollondilly, Wingecarribee, and Camden (*N* = 3) and from community health care workers in Fairfield and Liverpool (*N* = 2).

**Table 1:** Number of participants for each group and LGA.


LGA	COMMUNITY FORUMS:AGED CARE WORKERS	COMMUNITY FORUMS:OLDER PEOPLE	INTERVIEWS:GPS	TOTALS

Canterbury-Bankstown	8	8	2	*N* = 18

Camden	7	39	4	*N* = 50

Campbelltown	5	1	1	*N* = 7

Fairfield	10	6	1	*N* = 17

Liverpool	10	6	0	*N* = 16

Wingecarribee	12	15	4	*N* = 31

Wollondilly	8	11	2	*N* = 21

*Totals*	*N* = 60	*N* = 86	*N* = 14	*N* = 160


Gender breakdowns were as follows: GPs were 7 females and 7 males, community forums for older people were 63 females and 23 males, and for aged care workers were 57 females and 3 males. Separate community forums were attended by (1) older persons and their representatives, informal caregivers, and family members; and (2) aged care workers from public, private, and not-for-profit organisations including LGA council members, town planners, and policy-makers, home support and respite services, cultural and community associations and organisations, not-for-profit organisations, primary and community healthcare workers including community nurses and aged care assessment team members, hospital staff, residential aged care (aka nursing home or care home) service providers, and private health insurers.

### Thematic analyses

Four themes were identified across all data sources: (1) access to community aged care services, (2) healthcare and medical needs, (3) social concerns and needs, (4) education and information needs. Differences between LGAs are included in Supplementary Materials.

#### Theme 1: Access to community aged care services

Theme 1 had three sub-themes that are detailed in Table 2 with supporting quotes: 1.1 barriers to service access and utilisation, 1.2 ideas to facilitate service access and utilisation, 1.3 MyAgedCare government portal.

**Table 2:** Theme 1: Access to community aged care services.


SUB-THEMES	QUOTES

Barriers to service utilisation	*“It’s about being mobile, it’s about having access, it’s about who’s going to help you, who’s going to support you, who’s going to be an advocate for you. If you’re on your own and you are in your own home and you for some reason could not afford or weren’t aware that there were support services available for you, who’s going to help you?”* – Wingecarribee Community Forum Older People.

Ideas to facilitate service access and utilisation	*“Certain cultures will not necessarily go to a healthcare worker or a service for information because they may not be familiar with that service or they’re just not used to their country of origin having that service, so they would possibly go to … not a church leader, but someone that’s quite well-known in the community as like an elder.”* – Fairfield Community Forum Aged Care Workers.

MyAgedCare government portal	“*I just want to stress that CALD communities, they tend to have more difficulties, and the [MyAgedCare] call centre is a difficult place to go through if you’re from the CALD background, and as an assessor myself I’ve been reduced to tears trying to get through MyAgedCare the call centre. And then I’ve had beautiful experiences also where the person is like thank God you were on the phone today, thank you, can I call you at home, where are you in Australia, please I just want to talk to you. And then other times it’s like why?”* – Camden Community Forum Aged Care Workers.


*Note*: CALD = Culturally and linguistically diverse.

##### Barriers to service access and utilisation

There was considerable variation in participants’ perceptions towards the current number and types of available services. Barriers to utilisation and uptake (Table 2) included difficulty accessing due to location and transportation including community buses, long waiting times, lack of available information and awareness of services, cost, confusing referral pathways, system fragmentation, difficulty navigating for both healthcare workers and older people, and cultural and/or language disparities that may diminish understanding and utilisation of services. Participants noted some personal barriers including an unwillingness by older people to attend services due to feeling shy or uncomfortable or having difficulty accepting that they are ageing, and a lack of responsibility for one’s own health and ageing needs.

##### Ideas to facilitate service access and utilisation

Participants suggested bilingual health workers (rather than interpreters) and community leaders to enable older adults from culturally and linguistically diverse (CALD) backgrounds to understand, access, and utilise services (Table 2). Case managers to coordinate the access of services and help older people manage their health needs were considered important. Shorter wait times and reduced costs for services and treatments were suggested. Participants felt that it would be useful to have a one-stop-shop for aged care services offering different activities, such as tai chi, art classes, and education programs.

##### MyAgedCare government portal

Discussions regarding the national MyAgedCare government portal were generally negative, largely that it is confusing and difficult to navigate. This was not only seen as a problem for consumers with lower levels of information literacy and those from CALD backgrounds that may face other barriers, but also for those working in healthcare. Issues associated with MyAgedCare include the creation of referral problems such as longer wait times, the loss of referrals, and a lack of awareness and education surrounding the portal for the community and GPs.

#### Theme 2: Healthcare service and medical needs

Theme 2 comprised three sub-themes: 2.1 healthcare and medical issues for older people, 2.2 healthcare and medical issues for caregivers, 2.3 services needed; see Table 3.

**Table 3:** Theme 2: Healthcare service and medical needs.


SUB-THEMES	QUOTES

Healthcare and medical issues for older people	*“The staff aren’t really trained in aged care or dementia … there are inadequate registered nurses on duty.”* – Camden Community Forum Older People.

Healthcare and medical issues for caregivers	*“I think carers will regularly put their own health needs to the side to care for those that they’re looking after, especially if that person is at risk of going into residential care and they really don’t want that.” –* Canterbury-Bankstown Community Forum Aged Care Workers.

Services needed	*“I think it [improved access to allied health] would make a huge difference because it would reduce the … huge burden on general practice … and … reduce hospital admissions and … out of hours … as well.”* – Camden GP.


##### Healthcare and medical issues for older people

Dementia, diabetes, cardiovascular disease, cancer, arthritis, and mental health issues were perceived to be the most common medical issues that older adults face. Many other medical conditions including falls, asthma, macular degeneration, malnutrition, dental health, continence, hearing loss and acute conditions such as urinary tract infections (UTIs) were also listed, in addition to multi-morbidity-related complications. Participants felt that hospitals discharge elderly patients home too early and that there is a lack of outpatient care following hospitalisation, resulting in rapid deterioration following discharge.

Barriers to residential aged care facilities (RACFs) included costs, a lack of services, and trained staff (see Table 3). Participants perceived that RACFs, in general, are associated with a negative stigma or poor reputation, which consequently leads to many older people not wanting to relocate from their homes.

#### Healthcare and medical issues for caregivers

Neglecting one’s own health needs due to caring responsibilities was considered one of the prominent issues faced by informal caregivers. Many participants stated that caregivers often put all their time and effort into their caring role, forgoing their own medical appointments, leading to the carer becoming sick and sometimes even resulting in hospitalisation (Table 3).

A lack of respite was noted as a major healthcare issue for caregivers across all LGAs, which was linked with high stress levels, and physical and emotional exhaustion. Many caregivers faced negative financial impacts due to their caring responsibilities, mainly a loss of employment, income, and the provision of a small government allowance (Centrelink). More support, including education, in-home services, and finance was discussed.

##### Services needed

Participants described unmet needs for mental health, dementia, preventative health, and outpatients, and that more services were needed to address these issues. A lack of allied health professionals was discussed, particularly occupational therapists (OTs) and social workers. GPs felt that they would be able to better use their time if there was improved access to allied health, reducing hospital admissions and out of hours calls (Table 3).

#### Theme 3: Social concerns and needs

Table 4 details the three sub-themes and quotes for Theme 3: 3.1 social concerns for older people, 3.2 social concerns for caregivers, 3.3 ideas to address social needs.

**Table 4:** Theme 3: Social concerns and needs.


SUB-THEMES	QUOTES

Social concerns for older people	*“And you’ve got a high incidence of older people with depression, so this contributes to their social isolation as well. I think it’s just a vicious cycle for them.”* – Liverpool Community Forum Aged Care Workers.

	*“Social isolation, it makes older people more vulnerable … because if they’re spending a lot of their time at home and they don’t see anybody, it’s very easy for them to be approached by anyone and be financially abused because they’re lonely and they’re looking for friendship, and someone is suddenly being kind to them and paying them attention, so it opens them up to predators for I think financial abuse and other types of abuse as well.”* – Fairfield Community Forum Aged Care Workers.

Social concerns for caregivers	*“Well, there’s probably a lot of carer stress involved that they couldn’t get any respite … I’ve seen a lot of them become depressed and anxious about the situation, and in the end, they had to put them in the nursing home.”* – Fairfield GP

Ideas to address social needs	*“It’s time for older people to give something back …. Maybe just helping kids with homework, it can be heaps of things. So, older people should be encouraged to volunteer because it’s in giving that we actually receive.”* – Canterbury-Bankstown Community Forum Older People.


##### Social concerns for people

Social isolation was the most discussed social concern across all LGAs. Participants felt that social isolation occurs due to many factors including transportation issues (resulting in older adults becoming housebound), unwillingness to participate in social activities due to hearing or cognitive impairments, other disabilities, mental health issues (e.g., depression and anxiety), and loneliness caused by the loss of a loved one (Table 4).

Elder abuse, particularly financial, emotional and neglect, emerged as a common social concern, and was associated with stigma. Examples included family members borrowing money then not paying it back, and pressuring older parents to not sell their home to fund their needs so that a larger inheritance could be received. Table 4 details the account of one aged care worker who described the complex social precipitating factors. Emotional abuse included yelling at older parents leading to emotional and psychological harm. Examples of neglect included relatives not assisting their older family members to make decisions or supporting them to manage their healthcare adequately (e.g., visiting healthcare providers). Some participants felt that often the abuse is not malicious in nature, and stems from the busy schedules that families face.

Home maintenance needs including lawn mowing, gardening, cleaning, and ensuring all homes are easily accessible for those with mobility issues were listed as areas that required improvement. Participants felt that when these needs are not met, it may be difficult, dangerous, or embarrassing for older people to leave their homes or have visitors due to dwelling untidiness and disarray, resulting in further isolation. Other social concerns included the unaffordability of participating in social activities, cultural disparities, exclusion (particularly for those with dementia), lack of self-esteem, lack of social support groups, and underutilisation of technology (Table 4).

##### Social concerns for caregivers

Social isolation was commonly listed as a concern for informal caregivers resulting in risk of mental health issues, such as depression. A lack of time to build and maintain social relationships due to caring obligations, stress, isolation and loneliness, a lack of flexible respite services and social support programs were concerns, with one GP articulating the consequences (Table 4).

##### Ideas to address social needs

Participants suggested increasing the number of support groups and respite services for both older adults and informal caregivers, and improving access to social workers. Some participants felt that older people should be encouraged to participate in volunteer work as it is a free and fulfilling way to increase social activity, with the view of one older person shown in Table 4.

#### Theme 4: Education and information needs

Theme 4 covered four sub-themes shown in Table 5: 4.1 barriers to education and information access, 4.2 ideas to facilitate education and information access, 4.3 advance care planning education, 4.4 GP education.

**Table 5:** Theme 4: Education and information needs.


SUB-THEMES	QUOTES

Barriers to education and information access	*“Because you’re not computer minded, because you can’t transmit by message, because you can’t do technology … and I come from a migrant family that they never went to school and therefore I’m lucky enough that I can understand English … I haven’t got any of my family that can help me. I’m on my own, I need support.”* – Liverpool Community Forum Older People.

Ideas to facilitate education and information access	*“I think you also have to address their social and cultural contexts, because I have a lot of patients who I give them the information and I keep giving the information and we do it in all sorts of ways, but because they have these cultural beliefs that they can’t have people in their houses or you know, you can’t send your mum to a nursing home, there’s that breakdown.”* – Wollondilly Community Forum Aged Care Workers.

Advance care planning education	*“I think this advance care planning and the planning to die … needs to be talked about in the general community … advertisements, programs on TV [are] a great way to get the word out there to the mass public and start to talk about it, and the more you talk about it the less foreign it seems. I think that’s the only way, changing the way society thinks in general.”* – Liverpool Community Forum Aged Care Workers.

GP education	*“Because GPs are very time poor, because we deal with a broad spread of geriatric problems, training needs to be condensed. It needs to be clinically relevant, it needs to be basically concise and delivered in a way that the GP can then refer to sometime in the future, so it’s not the way the information is being delivered, [but] rather what sort of information we need.”* – Wingecarribee GP.


##### Barriers to education and information access

Many participants felt that older adults and their caregivers do not know where or how to access information about aged care services. Reasons discussed included perceived lack of education and information, inappropriate format (e.g., online only, difficult to comprehend/understand for those with disabilities), and that healthcare providers (e.g., GPs) lack the time and training to provide information, resulting in the underutilisation of aged care services. It was felt that information is confusing and difficult to navigate, sometimes only electronically, which could raise challenges for individuals with low levels of digital literacy, or those from CALD backgrounds when resources are not culturally or lingually appropriate (Table 5).

##### Ideas to facilitate education and information access

Participants commonly felt there was a need for more information and education about existing aged care services. Current education and information services were thought to be accessed via newspapers, online, the local council, at libraries, some GP offices, and via GPs directly. To increase awareness of available services, additional methods were suggested including media advertising (TV, radio, pamphlets), adverting at social outings, shopping centres, GP and hospital waiting rooms. Other ideas included providing information in hard copy and not just digitally, an older persons’ television channel, a helpline, and having the information available all in one location: an ‘information hub’ (either physical or virtual). The need for more culturally, ethnically, socially, and lingually appropriate education and information was articulated repeatedly across all LGAs (Table 5). Discussions centred on education services that provide information on elder abuse, financial matters, general health and wellbeing advice, and to reduce the stigma associated with ageing, encouraging uptake of aged care services and facilitating informed decisions about health and social needs.

##### Advance care planning education

Many participants noted that education on advance care planning is very important, but minimal planning is taking place within the community. Reasons included the sensitive nature of the topic, and perceived lack of acceptance and willingness to discuss planning for the future due to fear of sickness, disability, and death. It was suggested that the topic of death needed to be less taboo, and that education for advance care planning needs to begin earlier in life, provided by a mixture of professionals including GPs, nurses, specialists, Centrelink, and solicitors.

##### GP education

It was noted by some GPs that education regarding geriatric medicine, information and services needs to be short and concise (Table 5). Some GPs stated that they would like more training on diagnosing mental illnesses in older patients as it is easy to misdiagnose as dementia, and because sometimes patients are in denial or try to cover-up/downplay their symptoms, or resist a diagnosis. Some GPs felt that they were confident in the areas of falls identification and prevention, however, others felt that they would benefit from more education on this area as well as updates on available services. Several government-funded community initiatives where GPs access information were identified.

## Discussion

Over 21 months, 160 stakeholders including primary, acute, and community care providers together with policy makers, and consumers (i.e., older people), their informal caregivers and representatives provided key insights into the unmet needs of community aged care services in SWS, NSW Australia. This needs assessment identified key barriers and facilitators in relation to healthcare service access and utilisation, social concerns, and education and information for older people, caregivers, and healthcare providers. The approach was grounded by an action-oriented advocacy process for change with the aim of implementing findings – both identified problems and proposed solutions – to inform the planning of consumer directed models of care [5,6].

### Evaluation and Contextualisation of Findings

Most people prefer to ‘age in place’ and continue residing in their homes and communities, regardless of physical or mental changes [15]. There was perceived stigma regarding the transition to residential aged care, a finding that has been reported previously as “an evil to be avoided at all costs” despite the efforts of residential aged care staff to protect against stigma associated with seeking and accepting necessary care [16]. Promoting independence, conveying respect, supporting social engagement, and training staff to provide appropriate care have been suggested as appropriate mechanisms to reduce the stigma associated with the aged care sector [17]. Avoiding workforce shortages and enhanced training of the aged care workforce is an urgent international priority [18]. In Australia, the Royal Commission into Aged Care Quality and Safety identified a ‘workforce crisis,’ where 90% of aged care residents report a lack of staff to provide basic care [19]. Advance care planning also requires urgent attention across the healthcare sector [20], with large gaps in advance care directives present in hospital and primary care settings (Australian prevalence: RACFs at 47.7% hospitals at 15.7%, and general practice at 3.2%) [21].

There was significant burden associated with informal caregiving for older people resulting in unmet health and social needs for carers and reduced service and information access. Greater levels of caregiver burden are associated with older caregivers, longer durations of care, and poorer health status [22]. Community-based aged care services that can improve ADLs for older people and their caregivers may be associated with enhanced quality of life [23]. Assistive technology offers a potential avenue in reducing caregiver burden by increasing independence, saving time, reducing assistance, energy and workload, and improving safety [24]. It is essential that strategies are collaboratively planned and client-centred, given that the views of the most important factors for community living have been shown to differ between older people and their healthcare providers [25].

Discharge planning was identified as an urgent need to support older people transitioning from hospital back to the community to prevent deterioration and appropriately manage comorbidities in an integrated fashion. Factors that impact the effectiveness of discharge plans include the structure and organisation of the healthcare system, and the level of social support available including relationships with caregivers, healthcare providers, and education and information regarding self-care [26,27]. Discharge plans should have integrated input from the older person and their caregivers, and the multidisciplinary teams involved in the discharge and transition through acute, subacute, and community care settings including allied health workers, nurses, GPs, specialists, and preferably a care coordinator in order to bridge the gap between health and social care [28].

Social isolation and loneliness are associated with a raft of negative health and wellbeing outcomes for older people including 30–40% increased risk of mortality [29]. Around half of people aged 60+ years are at risk of social isolation, and a third have been estimated to experience loneliness [30], with these numbers increasing due to lockdown orders throughout the COVID-19 pandemic [31]. Risk factors for social isolation and loneliness include reduced mobility and income, family dispersal, losing loved ones, and poor health [32]. Here, we identified additional factors including being housebound, having a hearing impairment, an inability to connect through technology, cognitive and mental health issues including dementia and depression, and a lack of time for caregivers to build social connections. Interventions that have been employed with varying degrees of effectiveness to reduce social isolation and loneliness in older people include technology-based programs (such as video calls, help with computer use, or interaction with a companion robot), multi-strategy programs incorporating a social support component such as volunteering, and community programs such as engaged arts [33]. There are various opportunities and constraints linked with these different approaches, and it is important that such services and programs are co-created with the community to ensure their appropriateness for diverse populations. Others have called for a cultural-shift to viewing social isolation and loneliness as preventable, with the need to address these social factors across the lifespan [34].

Access to education and information in an appropriate format was considered a significant barrier for older people and healthcare providers. Unmet needs were identified for age-appropriate resources accessible to various CALD communities and catered for those with physical and cognitive disabilities. Bilingual and CALD aged care workers were recommended over interpreters as they can provide insights into culturally-appropriate, flexible, and person-centred care, particularly in the context of dementia [35]. Health literacy and education on the self-management of chronic conditions could be embedded into community aged care service delivery via telehealth [36], particularly for rural and remote communities. Despite healthcare workers reporting a need for more education, there is also a disconnect between perceived and actual knowledge and education requirements of health professionals. For instance, nursing staff report knowledge gaps, despite scoring well on gerontological nursing assessments [37]. Prior to investing in further educational services for healthcare providers, a knowledge assessment should be undertaken.

### Strengths and limitations

Strengths of this project include its large sample size, standardised data recording and transcription, reducing bias by having data collected by multiple researchers, and triangulation of data across multiple sources and geographical regions. Future research should also seek to triangulate data from a wider range of quantitative sources such as health service access, demand, and utilisation data and service mapping data.

Limitations include engaging with English speaking communities only, one LGA (Campbelltown) not being well-represented, particularly regarding the views of older people and their representatives, another LGA (Camden) being over represented (31% of all participants, and 45% of all community forums older people), and having more females than males for community forums (particularly aged care workers) across LGAs. The 21-months taken to recruit and collect data from GPs may mean that some issues had changed over the course of the study. Potential sampling bias may have been introduced due to the purposive sampling strategy where stakeholders specifically interested in community aged care needs were likely to come forward to participate, and confirmation bias based on the prompts used in community forums (see Supplementary Materials), however these were seldom required. Future research should also seek to triangulate data from a wider range of quantitative sources such as health service access, demand, and utilisation data, and service mapping data.

### Lessons Learned

- Using an applied, action-oriented advocacy process for change for this needs assessment was very successful and promoted community participation to identify their felt needs and propose solutions. Involving stakeholder groups from the outset in the needs assessments and scoping process, and later in service design and delivery ultimately increases buy-in, a sense of ownership, and service uptake and utilisation.
- Incorporating and triangulating the views of the broadest possible range of stakeholders from consumers through to policy makers is essential for increasing opportunities for care integration after needs assessment findings have been implemented.
- Create a single source of information for both consumers and healthcare providers. A ‘one-stop-shop’ – similar to [6] – can be the first point of access for older people to learn about their needs, empower them to take steps to address them and who to contact, and provide information on how to do this. By providing this information to healthcare providers as well, it will help to promote system integration by clarifying the roles and responsibilities of various providers.
- Upscaling and promoting central strategic services’ departments to coordinate and mitigate community issues would significantly avoid system fragmentation. For example, existing infrastructure in local government councils that links older people with transport, not-for-profit organisations, and health services could be upscaled and promoted widely to improve service integration.
- Upscale and integrate discharge planning between primary, acute, and community care, with a focus on support from allied health and GPs. This should align community demand with current and future hospital and health services and enable the development of a comprehensive and integrated care strategy to improve services for older people returning home from hospital.
- To successfully implement these findings into policy and practice, future work should seek to prioritise the needs reported here using a defined prioritisation technique such as the Hanlon method, multi-voting technique, nominal group technique, prioritisation matrix, or strategy grids [38]. Such a mechanism can provide an objective structure to practically rank needs and facilitate decision-making within the specific context of this study and ultimately improve health care service quality and safety.

## Conclusion

This case study showcases the process undertaken to identify community care needs for older people in SWS Australia from strategy, policy, and service delivery perspectives. Gaps have been identified in the strategic approach to integrated healthcare service delivery by the primary stakeholders including older people, their caregivers and representatives, GPs, specialists, allied health workers, and community aged care providers across the seven LGAs that comprise the region. Key solutions were identified by stakeholders to address the gaps in the medical, social, and education needs of older people in this highly diverse, complex, and high growth region of Sydney. The key learnings from this case study are intended to inform and guide future international needs assessments in addition to informing local policy, strategies, and service delivery of community health and education programs for older people that are essential for an effective integrated healthcare system.

## Additional File

The additional file for this article can be found as follows:

10.5334/ijic.7005.s1 Supplementary Materials. Supplementary Materials contains information on the study setting context, the interview schedules employed, and findings from differences between local government areas.
