# Supplementary material for: “I’m On My Own, I Need Support”: Needs Assessment of Community Aged Care Services
Source: Int J Integr Care. 2023 Sep 22;23(3):14. doi: 10.5334/ijic.7005 (PMC10516138; doi:10.5334/ijic.7005)
Supplement: Supplementary Materials. — Supplementary Materials contains information on the study setting context, the interview schedules employed, and findings from differences between local government areas. [file ijic-23-3-7005-s1.pdf]

Supplementary Materials

Introduction

Study Context

South Western Sydney (SWS) houses 12.3% of the Australian New South Wales (NSW) population across seven local government areas (LGAs) (NSW Department of Health, 2020). The region ranges from rural to metropolitan, and its residents are demographically, ethnically, and socioeconomically diverse. SWS has a high population growth rate that is 168% above the NSW average (2.7% vs. 1.6%) (NSW Department of Planning & Environment, 2015), and comprises some of the largest and fastest growing LGAs in Australia (Australian Bureau of Statistics, 2019, 2020). The region is culturally and linguistically diverse (CALD), with three of the most densely populated LGAs having high rates of people born overseas (40–54%; Canterbury-Bankstown, Fairfield, and Liverpool) (Australian Bureau of Statistics, 2016), and many residents speaking a language other than English at home (e.g., 60% in Canterbury-Bankstown). Together, the high population growth rates, CALD communities, and ageing population have implications for long-term planning of community health services in SWS.

Methods

Interview Schedule: GPs

ITEM 1 – Understanding our current position

1.1 What is your perception on what healthcare services for seniors exist in the community at this point?

Prompts:

- What's out there? Is this enough? Why/Why not?

1.2 Does the source of funding for seniors' healthcare impact how you can deliver care?

ITEM 2 – Healthcare and medical needs of seniors

2.1 What are the main healthcare issues you perceive for seniors in the region? Why?

Prompts:

- Access to information and services?
- Availability of specialists?
- Level of care in nursing homes?

2.2 What are the main healthcare issues for carers in the region? Why?

2.3 What is the most important medical issue you feel seniors face? Why?

2.4 Is there anything that your geriatric patients need that they are not getting?

2.5 In your opinion, what is the biggest barrier to your meeting the healthcare needs of your elderly patients?

2.6 What tools do you use to access information about services relevant to your geriatric patients?

2.7 What education or training do you think GPs would value in geriatrics?

2.8 What issues, if any, have you experienced with diagnosing mental illness in geriatric patients?

2.9 Are you confident in the area of falls cause identification & prevention?

2.10 Can the PHN assist you with your care to seniors, and how?

ITEM 3 – Social needs of seniors

3.1 What are the main social concerns seniors face in the region?

3.2 What impact does a lack of community social workers have for your and the management of your seniors?

ITEM 4 – Education and information needs of seniors

4.1 What types of education and information services are there available for seniors in relation to healthcare services?

ITEM 5 – Options for the future

5.1 Where do you think the greatest need is for the elderly – In medical management, addressing social problems, or education and information?

5.2 What medical management problems are the highest priorities to address?

5.3 What social problems are the most important to address first?

5.4 What education and information issues are the most important to address first?

5.5 Are there any local unique issues which could impact the quality or delivery of healthcare to seniors you assist?

5.6 Is there anything else relevant to the healthcare needs of elders in this region that we have not covered?

Focus Group Schedule: Community Forums Older People

ITEM 1 – Understanding our current position

1.1 What is your perception on what healthcare services for seniors exist in the community at this point?

Prompts:

- What's out there? Is this enough? Why/Why not?

1.2 Where does the funding come from to run community healthcare services for seniors in Australia and NSW?

Prompts:

- Federal, state, or local government? Privately funded?

ITEM 2 – Social or logistic needs and concerns of seniors

2.1 What are the main social problems seniors face in the region?

Prompts:

- Transport
- Elder abuse
- Accommodation/Looking after home

- Social isolation

2.2 What are the main social concerns carers face in the region?

ITEM 3 – Healthcare and medical needs of seniors

3.1 What are the main healthcare issues for seniors in the region? Why?

Prompts:

- Access to information and services
- Availability of specialists
- Level of care in nursing homes
- Cost of healthcare

3.2 What specific challenges do carers face to maintaining their own health

3.3 What is the single most important medical issue you feel seniors face? Why?

Prompts:

- Diabetes and its complications: is it a significant problem for the elderly or their carers to manage
- Dementia
- Falls
- Mental health – depression
- Continence
- Mobility
- Vision and hearing

ITEM 4 – Education and information needs of seniors

4.1 What types of health education and information services are there available for seniors in our region?

4.2 Where and how can this information be accessed? Where would you like to see this information accessed?

4.3 With regards to future healthcare needs, do you think older adults and carers have a reasonable understanding and ability to plan for this?

4.4 Do you as patient representatives, feel the new MyAgedCare government portal has made it easier for you to understand how to access aged care services?

ITEM 5 – Options for the future

5.1 Where do you think the greatest need is for the elderly – In medical management, addressing social problems, or education and information?

5.2 What medical management problems are the highest priorities to address?

5.3 What social problems are the most important to address first?

5.4 What education and information issues are the most important to address first?

5.5 Considering planning for the future (Advance Care Planning), do you feel the elderly, families and carers would want to discuss this with their long-term GP, a specialist, both or someone else? Why?

Prompts:

- A nurse specialist
- A private provider

Focus Group Schedule: Community Forums Aged Care Workers

ITEM 1 – Understanding our current position

1.1 What is your perception on what healthcare services for seniors exist in the community at this point?

Prompts:

- What's out there? Is this enough? Why/Why not?

1.2 Where does the funding come from to run community healthcare services for seniors in Australia and NSW?

Prompts:

- Federal, state, or local government? Privately funded?

ITEM 2 – Healthcare and medical needs of seniors

2.1 What are the main healthcare issues for seniors in the region? Why?

Prompts:

- Access to information and services?
- Availability of specialists?
- Level of care in nursing homes?

Select 5 most frequently mentioned issues and have participants allocate hypothetical budget to addressing this issue (%).

2.2 What are the main healthcare issues for carers in the region? Why?

2.3 What are the most important medical issue you feel seniors face? Why?

Prompts:

- Diabetes and its complications: is it a significant problem for the elderly or their carers to manage?
- Dementia
- Falls
- Mental health – depression
- Continence??
- Mobility?
- Vision and hearing

ITEM 3 – Social needs of seniors

3.1 What is the main social concerns seniors face in the region?

Prompts:

- Transport
- Elder abuse

- Accommodation/Looking after home
- Social isolation

3.2 What is the main social concerns carers face in the region?

ITEM 4 – Education and information needs of seniors

4.1 What types of education and information services are there available for seniors in relation to healthcare services?

4.2 Where do seniors and their carers access education and information services? Where would you like to see this information accessed?

4.3 Do you think older adults and carers have a reasonable understanding and ability to plan for their future healthcare needs?

4.4 Do you as patient representatives, feel the new MyAgedCare government portal has made it easier for you to understand how to access aged care services?

ITEM 5 – Options for the future

5.1 Where do you think the greatest need is for the elderly – In medical management, addressing social problems, or education and information?

5.2 What medical management problems are the highest priorities to address?

5.3 What social problems are the most important to address first?

5.4 What education and information issues are the most important to address first?

5.5 Considering Advance Care Planning, do you feel the elderly, families and carers would want to discuss this with their long-term GP, a specialist, both or someone else? Why?

Prompts:

- A nurse specialist?
- A private provider?

Results

Differences between LGAs

Differences between the 7 LGAs comprising SWS were explored. Camden was perceived to lack local senior-specific healthcare services, local specialists, dementia-specific services, and GPs. Long wait times were also flagged, which were anticipated to worsen as the area's population expanded. Canterbury-Bankstown was noted to have a large population of older people from CALD backgrounds who had strong family support, but also experienced language barriers to accessing services, malnutrition, and that more culturally appropriate mental health services were needed. Campbelltown was discussed as having a lack of outpatient services and specialists for seniors, and that gambling was a large social problem for this area. Fairfield was flagged as lacking many aged care services, despite being a relatively large LGA, particularly community palliative care services, multidisciplinary team access, and case management, in addition to appropriate housing facilities, particularly older people from CALD backgrounds due to the high refugee intake in the region. Liverpool was noted to have a good number of services, however some of those were not accessible to CALD communities. There was also the perception that a high proportion of older people in Liverpool come from low socioeconomic backgrounds without private health cover, further complicating health service access. Due to their rural and remote locations, Wingecarribee and Wollondilly were both discussed as having minimal services and that transportation options were lacking, making service access in other locations challenging and increasing social isolation. Wingecarribee was also noted to have good information sharing, particularly in relation to falls prevention programs and chronic disease education forums.

References

Australian Bureau of Statistics. (2016). *Census of Population and Housing*. Retrieved from Canberra:

Australian Bureau of Statistics. (2019). *Regional Population Growth, Australia, 2017-2018*. Retrieved from

Australian Bureau of Statistics. (2020). *Regional Population Growth, Australia, 2018-19* (3218.0). Retrieved from

NSW Department of Health. (2020). South Western Sydney. Retrieved from https://www.health.nsw.gov.au/lhd/Pages/swslhd.aspx#:~:text=South%20Western%20Sydney%20Local%20Health,population%20of%20approximately%20966%2C450%20people.

NSW Department of Planning & Environment. (2015). *NSW Population, Household and Dwelling Projections, 2014-2015*. Retrieved from
